# Biophysical properties of presynaptic short-term plasticity in hippocampal neurons: insights from electrophysiology, imaging and mechanistic models

**DOI:** 10.3389/fncel.2014.00141

**Published:** 2014-05-22

**Authors:** Ranjita Dutta Roy, Melanie I. Stefan, Christian Rosenmund

**Affiliations:** ^1^Department of Medicine Solna, Karolinska InsitutetStockholm, Sweden; ^2^Neuroscience Research Center (NWFZ), Charite UniversitatsmedizinBerlin, Germany; ^3^Department of Neurobiology, Harvard Medical SchoolBoston, MA, USA

**Keywords:** short-term plasticity, vesicle dynamics, hippocampal neurons, biophysical models, imaging, electrophysiology

## Abstract

Hippocampal neurons show different types of short-term plasticity (STP). Some exhibit facilitation of their synaptic responses and others depression. In this review we discuss presynaptic biophysical properties behind heterogeneity in STP in hippocampal neurons such as alterations in vesicle priming and docking, fusion, neurotransmitter filling and vesicle replenishment. We look into what types of information electrophysiology, imaging and mechanistic models have given about the time scales and relative impact of the different properties on STP with an emphasis on the use of mechanistic models as complementary tools to experimental procedures. Taken together this tells us that it is possible for a multitude of different mechanisms to underlie the same STP pattern, even though some are more important in specific cases, and that mechanistic models can be used to integrate the biophysical properties to see which mechanisms are more important in specific cases of STP.

## Dynamics of STP in hippocampal neurons and the role of biophysical models

Heterogeneity in synapse architecture and the frequencies at which synaptic information is transmitted cover a wide range in nature and depend on the task of the synapse. Short-term plasticity (STP) manifests itself as a change in post-synaptic current amplitude as a consequence of recent activity in a synapse. In the hippocampus, synapses that are relatively small and are involved in explicit and spatial memory transmit information in short bursts of action potentials at frequencies of tens of Hz. In contrast, the Calyx of Held, a large inner ear synapse, can transmit at up to hundreds of Hz. STP can be classified based on its directions and its kinetics. The short-term forms of STP are facilitation and depression. Facilitation is seen when the synapse increases its release over time, and depression is seen when this decreases. The generally accepted hypothesis to explain facilitation is increased residual calcium at the vesicular release site, and depression is often associated with depletion of vesicles (Zucker et al., 2002). The other types of short-term plasticity kinetics are enhancement on longer time scales. These include augmentation which lasts 5–10 s and post-tetanic potentiation which lasts up to 30 s. Because of the need of good temporal resolution, STP is often studied by electrophysiology. However, imaging techniques are also often used.

Neurotransmitter release occurs within milliseconds of action potential invasion into the presynaptic neuron. Once the axon has been depolarised, voltage-gated calcium channels open giving rise to presynaptic calcium influx (Bischofberger et al., 2002). The calcium binds to fusion sensors of vesicles that are docked at the release sites in the active zone and have been primed for release. This makes the vesicles fuse with the plasma membrane with the help of the SNARE complex and its associated proteins. Upon fusing they release neurotransmitters into the synaptic cleft, which diffuse and bind to post-synaptic receptors, and are removed from the cleft by buffering and diffusion. Neurotransmitter release is governed by the life cycle of synaptic vesicles that carry neurotransmitters in the synapse, and their probability of release is a key factor (Katz and Miledi, 1963; Heuser and Reese, 1973; Heuser et al., 1979). Each step of the life cycle of an individual vesicle is controlled by molecular regulators, and the activities of the fusion sensors are largely dependent on calcium (Katz and Miledi, 1968; Rosenmund et al., 2003; Rizo and Rosenmund, 2008). Different imaging techniques can be used to look directly at underlying global calcium dynamics and vesicle movement. To sum up, short-term plasticity is a consequence of the structure of the synapse, the architecture of the active zone itself, the concentration profiles of presynaptic calcium, the vesicular release probability and the kinetics of neurotransmitters in the cleft and the gating of post-synaptic receptors. Due to the short time frame and the complexity of the mechanisms underlying synaptic release, differences in physical properties of the release machinery are likely to have a large impact on the pattern of short-term plasticity. Understanding how the underlying heterogeneity in structure and biophysical properties of the synapse gives rise to heterogeneity in short-term plasticity can help us to understand how different synapses work and what is changed in pathological states of the hippocampal pathways such as memory impairment (Wood et al., 2000; Froc et al., 2003; Neves et al., 2008; Witton et al., 2010; Holderith et al., 2012).

Because of technical limitations, the kinetics and quantitative properties of all the molecular and biophysical mechanisms for changes in release patterns cannot be monitored experimentally at the same time as STP is measured. Therefore, in order to elucidate the causes and their interactions, to understand disparate data and to be able to make predictions about STP and evoked release, mechanistic mathematical models have been formulated over the years for different types of synaptic boutons (Trommershä user et al., 2003; Pan and Zucker, 2009; Nadkarni et al., 2010). These models have been constructed to complement experimental data by giving insight into interactions of the components underlying neurotransmitter release and short-term plasticity, and analyzing it at a deeper level. They are formulated with strong support from electrophysiological and biochemical data, and are usually validated, which supports the predictive ability and usefulness of the approach.

**Figure 1:**
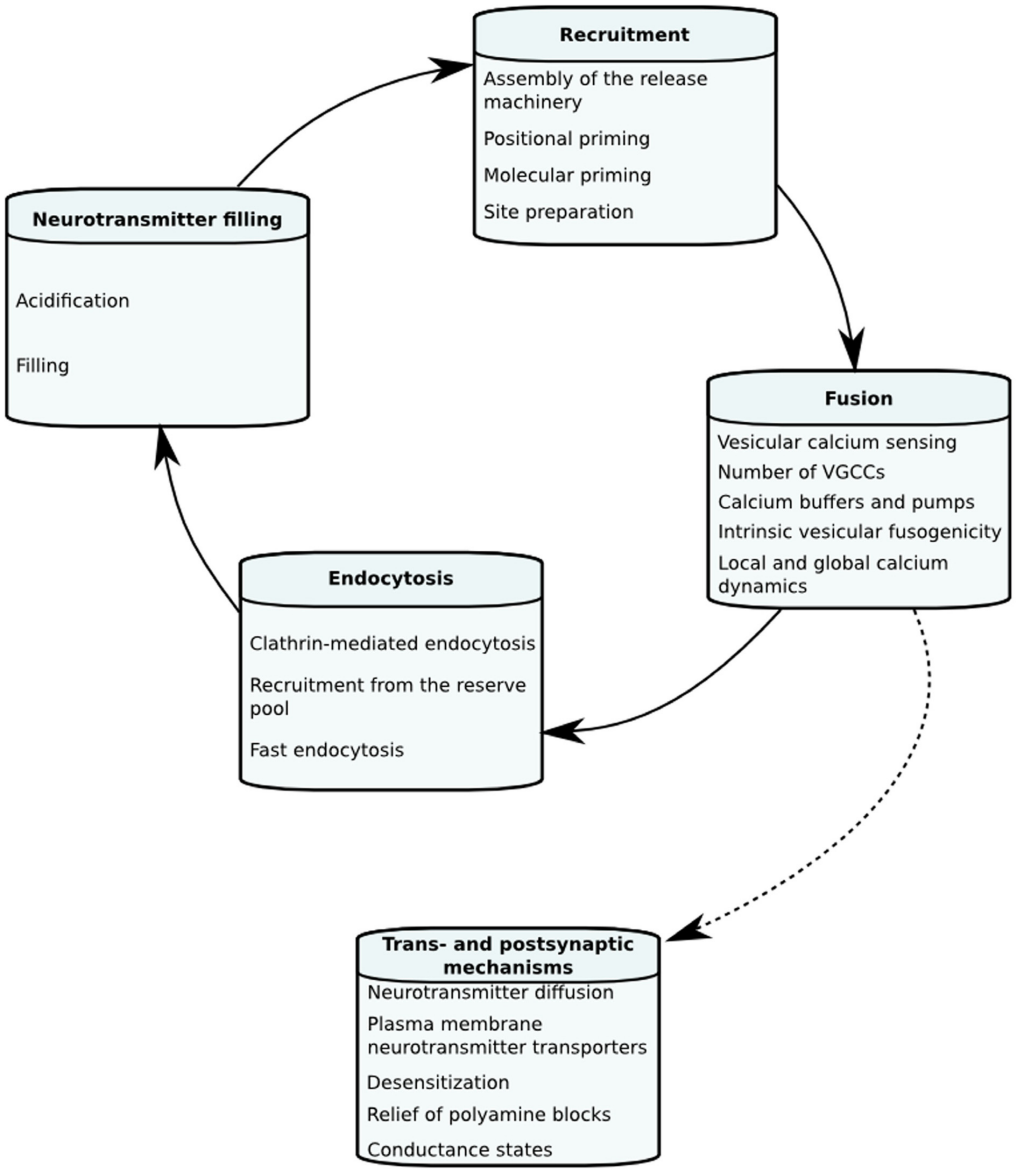
**Important factors in the stages of short-term plasticity**.

The goal of this review is to look into biophysical mechanisms underlying STP and what dynamical and quantitative information imaging, electrophysiology and mechanistic models have given about neurotransmitter release and STP in hippocampal neurons. We, however, also discuss some data and models from other systems. In the first section we will define mechanistic models of neurotransmitter release and STP in order to give the reader a picture of how they are constructed and how tightly linked they are to experimental findings. Following this we will go through individual biophysical steps of the vesicle life cycle, and their kinetic and quantitative properties that can change the dynamics of neurotransmitter release and STP in hippocampal synapses. The different factors affecting each stage of STP are illustrated in Figure 1.

## Constructing mechanistic models of STP

**Figure 2:**
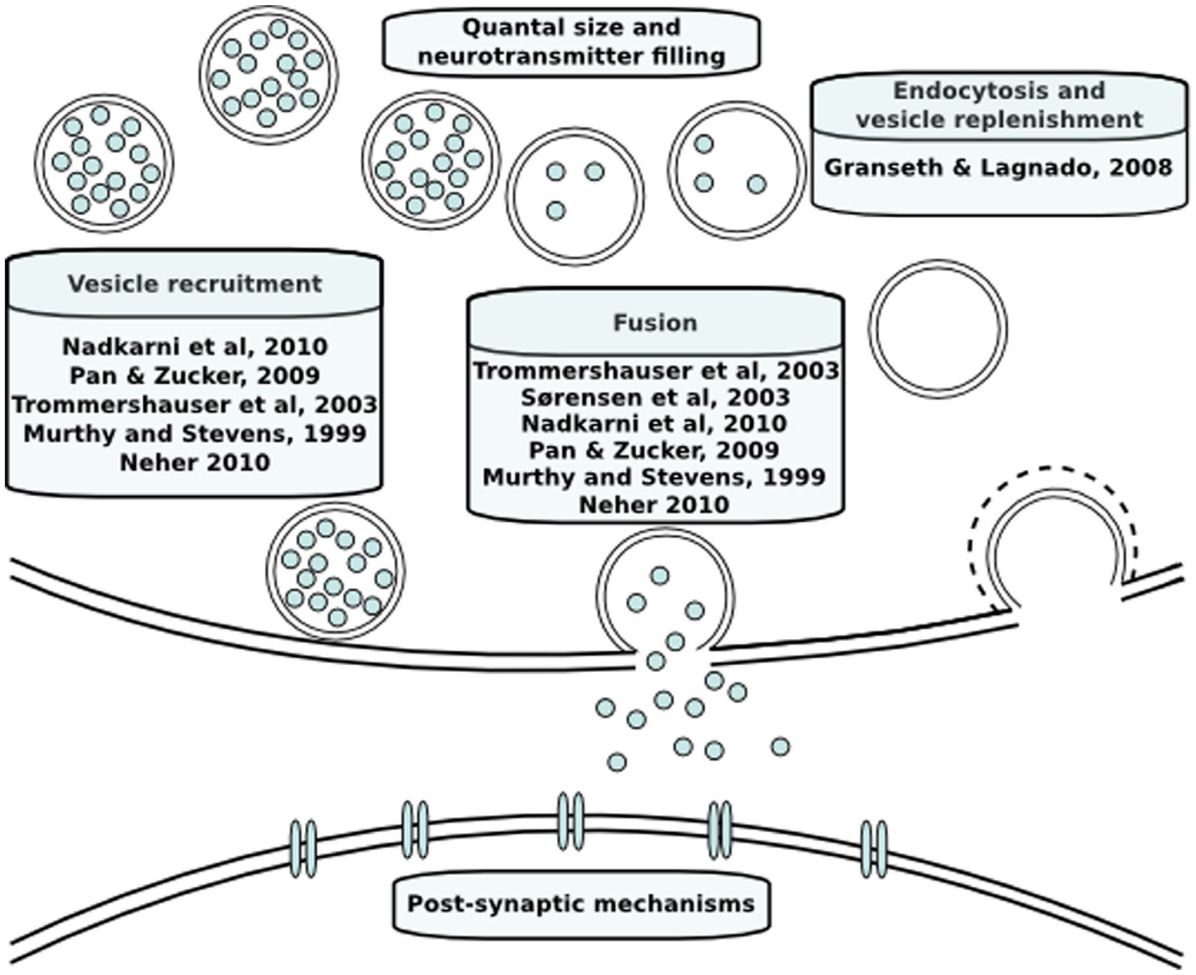
**Modeling papers discussed here in their functional context**.

Neurotransmitter release has been modeled both phenomenologically and mechanistically in the past. An overview of the models discussed in this review is provided in Figure 2. In the case of phenomenological models, the aim is to integrate data and explain phenomena with a quantitative model without in-depth mechanistic explanations. In contrast, the aim of mechanistic models is to investigate the plausibility of specific mechanisms to explain phenomena (Rodrigue and Philippe, 2010). The models we will discuss below are predominantly mechanistic and describe dynamics of neurotransmitter release with a set of coupled reactions that describe the kinetics of causal intracellular processes, and are either spatially explicit or assume a homogeneous distribution of molecules in space. Reaction rates of these processes are formulated based on laws of chemical reaction kinetics. Therefore, in order to construct a model, values of the parameters such as rate constants or initial concentrations of vesicles or sites need to be determined. Parameter determination is commonly done by identifying single values of parameters by literature search. Despite their biological relevance, there is a paucity of kinetic data on the life stages of a vesicle and local calcium at the release site due to technical limitations. In those cases, mathematical optimization is necessary to constrain unknown parameters using knowledge about the expected behavior of neurotransmitter release. Moreover, different conditions that are not taken into account in the model will induce a change in the dynamics of individual intracellular mechanisms and different combinations of their dynamics can theoretically produce the same short-term plasticity behavior. For instance, there is a temperature sensitivity of the refilling rate of releasable vesicles, which is a component that is seldom taken into account (Pyott and Rosenmund, 2002). A more detailed discussion of parameter estimation in order to overcome this problem has been done elsewhere (Locke et al., 2008). Whether or not this problem is taken into account, a first step prior to mathematical parameter estimation is to constrain the parameters to a biologically realistic order of magnitude. Such approximations are done based on quantitative and kinetic data from experiments that we will go through below.

## Recruitment of synaptic vesicles to their release sites

To prepare for fusion with the plasma membrane the vesicle needs to become positionally and molecularly primed (Neher, 2010). Positional priming means that the vesicle docks at a release site that will enable it to sense calcium upon influx from voltage-gated calcium channels or other types of calcium channels like the P2X2 ATP receptors (Kaeser and Regehr, 2014). The basic definition of a docked vesicle is when the vesicle is in the active zone. This area is electron dense and can therefore be detected by electron microscopy (EM). In the past, the distance between a docked vesicle and the plasma membrane has been measured to tens of nm by EM (Heuser and Reese, 1973; Heuser et al., 1979; Toonen et al., 2006). However, modern ultrastructural analysis has shown that docked vesicles are situated directly at the membrane (Watanabe et al., 2013). In addition to physical vicinity to the membrane the vesicle also needs to go through a series of molecular changes in order to become primed for release. Molecular priming is what prepares the vesicle to overcome the energy barrier for fusion and is defined electrophysiologically by the ability of a vesicle to fuse upon hypertonic sucrose stimulation at a concentration of 500 mM or upon high frequency stimulation (Rosenmund and Stevens, 1996; Murthy and Stevens, 1999). This step is often assumed to bring the docked vesicle even closer to the plasma membrane, for instance by tightened physical coupling of the vesicle release machinery consisting of the SNARE complex and its associated proteins. It is clear that priming is a physically separate step, since the readily releasable pool varies upon manipulation of priming proteins (Rosenmund et al., 2003). However, the relationship between docking and priming does not necessarily have to be linear since molecular priming might also occur upstream of docking (Verhage and Sörensen, 2008).

The size of a vesicle is around 40 nm, which is substantial compared to the dimensions of small hippocampal synapses. Moreover, since vesicles are linked with the cytoskeleton, the vesicle will use mechanisms to move toward its release site in the plasma membrane that are not only dependent on free diffusion. In hippocampal neurons, for instance, it has been suggested that the slow pool is recruited to the fast pool in an actin-dependent manner (Lee et al., 2012). The mobility of vesicles is implicit in the docking rate in models where spatial distribution is not taken into account (Pan and Zucker, 2009). The basal docking and priming rates can be constrained by looking at the recruitment rate of the readily releasable pool after depletion with high frequency stimulation or application of hypertonic solution (Pyott and Rosenmund, 2002). In autaptic hippocampal cultures stimulated by 500 mM sucrose, the RRP was recruited with a time constant of 0.49 s for the slow releasing pool and 4.28 s for the fast releasing pool under physiological temperatures, which is three times faster than at 25°C. In high-pressure freezing electron microscopy experiments, the rate of replenishment of vesicles docked at the membrane was similar to that of the RRP (Watanabe et al., 2013). The recruitment rate of the readily releasable pool is dependent on the global calcium concentration in the presynaptic bouton and therefore increases over trains of stimulation (Neher and Sakaba, 2008). The question of the impact of molecular priming rate under short-term stimulation was investigated in a comprehensive model of tonic and phasic synapses in crayfish neuromuscular junction. In this model, varying only the priming rate while keeping other parameters constant could reproduce the depressive and facilitating behavior seen in these two different types of synapses as well as differences in asynchronous and synchronous release between them (Pan and Zucker, 2009). In a model of the Calyx of Held, it was predicted that these synapses could not facilitate without calcium dependency of the recruitment rate (Trommershä user et al., 2003). Docking of vesicles is also known to be reversible, and the time constant for the backward reaction has been estimated from simple models of vesicular release (Murthy and Stevens, 1999; Neher, 2010).

Not only the kinetics of docking and molecular priming are important for short-term plasticity, but the proportion of docked and molecularly primed vesicles under resting conditions will also affect the direction in which the STP curve will go initially. Electron microscopy data from hippocampal autaptic cultures showed that under basal calcium concentrations, the numbers of morphologically docked vesicles were similar to the numbers of readily releasable vesicles measured electrophysiologically by depleting the pool with high frequency stimulation. This suggests that most docked vesicles are molecularly primed under steady-state (Murthy and Stevens, 1999). The authors of that paper had also assessed the total recyclable pool using FM-dye and estimated the total recyclable pool to be around three times the size of the readily releasable pool.

So far we have seen that proper assembly of the release machinery is required for vesicles to dock and become molecularly primed. However, the sites at which they dock also have a unique molecular character and can display dynamics that can be relevant for STP (Boyken et al., 2013). In the past it has often been assumed that the number of release sites is constant and that the only mechanism responsible for steady-state release is the balance between depletion of vesicles and calcium dynamics. According to the “slot hypothesis,” a rate-limiting step could be retrieval of the proteins from the fused vesicles in order to reuse them for the sites (Neher, 2010). The existence of this phenomenon and its impact on STP has been tested by knocking out proteins that are endocytosed with the vesicle. Hence, the number of sites that can be used does not remain constant. The impact of refractory time for release from the same bouton was tested in a spatially explicit model of a single hippocampal bouton and release site (Nadkarni et al., 2010). It was shown that at high release probabilities, assigning a short refractory time to release from the same bouton, gives a post-synaptic current behavior that is closer to experimental release rates from evoked release. Mechanistic evidence from hippocampal cultures suggests that sites need to be cleared and undergo priming prior to vesicle docking, which implies that the number of release sites varies over time and that the priming of sites will require a certain refractory time, supporting the role of site-priming in short-term plasticity (Neher, 2010). The concept of refractory time, however, is not only related to the lack of release sites, but may also be related to the biophysical reluctancy of the cell membrane to fuse with vesicles directly upon a previous event as discussed below.

## Vesicle fusion

Fusogenicity of the vesicle is dependent on how fit the individual vesicle is to overcome the energy barrier for fusion, which is determined by its state of molecular priming, but also by where the vesicle is situated when the action potential comes in, which is referred to as positional priming as we mentioned earlier (Neher, 2010). Both of these factors contribute to how fit the synapse is to release vesicles as a whole. Fusion can be either spontaneous or elicited by an action potential. It is often debated what the underlying determinants of action potential evoked release probability and spontaneous fusion are, and why there is a heterogeneity of release probabilities between different synapses and vesicles (Hanse and Gustafsson, 2001). Action potential evoked neurotransmitter release can exhibit different time scales with respect to how soon after depolarization release occurs and is therefore classified as synchronous or asynchronous release (Goda and Stevens, 1994). The former exhibits time scales of ms, whereas the latter exhibits time scales of tens of ms (Scheuss et al., 2007). The release probability upon action potential stimulation is known to be highly dependent on local calcium concentration at the active zone, whereas spontaneous release has shown a lose link with calcium (Wasser and Kavalali, 2009; Vyleta and Smith, 2011). Is the heterogeneity in release probability then mainly due to (1) different types of calcium sensors, (2) the amount of voltage-gated calcium channels at the site of release, (3) calcium handling mechanisms or (4) the calcium independent intrinsic properties of the vesicle that we referred to as molecular priming earlier?

The identity of the calcium sensors for asynchronous and synchronous release has been a long standing debate. The calcium sensors for fast synchronous release in hippocampal neurons are synaptotagmin 1 and 2, which are low affinity calcium binding molecules with five calcium binding sites (Walter et al., 2011). This was tested early with the help of a mechanistic model for chromaffin cells, which changed the affinity of synaptotagmin to calcium from its wild-type state and was able to fit the mutant behavior (Sörensen et al., 2003). However, the calcium sensor for slow release is still being speculated on Sun et al. (2007).

Since release probability is calcium dependent, one would expect that vicinity to calcium channel clusters through which calcium flows into the cell upon depolarization, will give higher release probabilities. The question of whether differences in release sites could be a factor in heterogeneity of release probability was addressed in a model of the Calyx of Held, where the docking sites were thought to be diverse, with some of them containing calcium channel clusters and others lacking them. The presence or absence of calcium channel clusters affects the amount of local calcium at different sites and thereby the release probability (Trommershä user et al., 2003; Hefft and Jonas, 2005). The model could only show a sustained steady-state release when the authors introduced a heterogeneity in release probability without which the vesicles were completely depleted, suggesting that heterogeneity can be due to different structural properties of release sites.

After influx of calcium from voltage-gated calcium channels, the synaptic bouton takes care of the calcium either by buffering or by pumping it out. Another possibility is calcium induced calcium release from intracellular stores, but it has not been implicated in STP (Carter et al., 2002; Wan et al., 2012). The buffering can be mediated by mitochondria or calcium buffers like calbindin in hippocampal synapses, where the former are implicated in short-term plasticity, but the latter is under debate (Jouvenceau et al., 1999; Wan et al., 2012). The extrusion can be mediated by plasma membrane ATPases (PMCAs) or sodium calcium exchangers, but the latter have low affinity for calcium. Like everything else involved in synaptic transmission, buffers and pumps also have their own dynamics. For instance, calcium buffers are thought to reach a certain degree of saturation over a train of stimulation (Berggård et al., 2002; Felmy et al., 2003). On the other hand, PMCAs are known to increase their affinity for calcium up to 20-fold upon interaction with calcium bound calmodulin (Brini, 2009). Calcium handling evidently affects short-term plasticity and due to its complexity as just discussed, a lot of insight could be gained from modeling it. Buffering, pumping and diffusion of calcium have been taken into account explicitly in a model of a single hippocampal synapse, where the authors found that spatial coupling between the voltage-gated calcium channels and the vesicles plays a major role in how fit the synapse is to release vesicles as well as how long it takes for the synapse to release upon an action potential (Nadkarni et al., 2012). This shows why calcium diffusion as well as extrusion and influx mechanisms are important in shaping the plasticity pattern of the synapse.

The dependence on local calcium of action potential evoked release can be shown by fast and slow binding calcium buffers. At distances below 100 nm only the fast calcium chelator BAPTA can bind the calcium, but at higher distances the slow chelator EGTA can also bind (Eggermann et al., 2012). The global underlying synaptic calcium levels in cortical pyramidal neurons lie around 100 nM at basal conditions and increase by 500 nM upon action potential stimulation (Koester and Sakmann, 2000). The concentration of calcium localized next to the membrane has been measured to be around 200 nM using membrane bound calcium dyes under resting conditions in skeletal muscle cells and rod bipolar neurons (Bruton et al., 1999; Wan et al., 2012). The rise time for the global calcium lies on the time scale of ms and the decay time has been measured to 0.1 s in cortical pyramidal cells (Koester and Sakmann, 2000). The near-membrane calcium residual and decay time have been estimated to submillisecond scales in the Calyx of Held (Meinrenken et al., 2003).

In the Calyx of Held, which is a synaptotagmin dependent system just like hippocampal synapses, calcium uncaging experiments have been done in order to investigate the local calcium concentrations required for vesicle fusion (Schneggenburger et al., 2002). These showed that saturation for the release rate is reached at 25 μM. Nevertheless, fusion rates similar to wild-type levels are reached already at 10 μM and the authors therefore suggested that fusion takes place below saturation of the synaptotagmin binding sites under normal conditions. Release probability can be described as dependent on the local calcium concentration with a Hill cooperativity constant for calcium binding, which has been estimated to 4 in the Calyx of Held (Neher and Sakaba, 2008). Hippocampal boutons are small in size and the possibility to do calcium imaging is therefore limited, but we can use the values from the Calyx as pointers. Unfortunately, whole-cell recordings from Calyx in combination with imaging show that there is no easy way of deducing the internal global synaptic calcium concentration from the external concentration, since there is a non-linear relationship between the two (Dodge and Rahamimoff, 1967; Schneggenburger et al., 1999). About the direct relationship between external calcium and local calcium nothing is known.

All three groups of determining factor types for action potential evoked release probability discussed thus far are calcium dependent. The dynamic regulation of calcium shows us why it is so important where the vesicle is situated when the action potential comes in, as it will have a stronger effect in the vicinity of calcium channel clusters. In addition to positional priming, we have also mentioned that the molecular priming state of the vesicle is important for the fusogenicity of the vesicle. The molecular priming mostly includes calcium independent mechanisms. A major contributor to the molecular priming state is clearly the setup of release machinery associated proteins, such as the presence of complexin, which facilitates fusion under basal and increased levels of calcium, and is therefore hypothesized to regulate vesicle fusogenicity in a calcium independent way (Xue et al., 2008). Some calcium independent factors will be specific to the vesicles themselves such as differential expression of surface proteins or membranes (Takamori et al., 2006). Membrane properties inherent to the vesicles and the plasma membrane can also affect the fusogenicity of vesicles in a specific bouton. For instance, it has been seen that the amount of cholesterol in a cell as well as the presence of certain phospholipids in the cell membrane such as *PIP*_2_ can enhance fusogenicity of vesicles (Martin, 2001; Rohrbough and Broadie, 2005; Wasser and Kavalali, 2009; Shin et al., 2010). In neuroendocrine cells secretory vescles can for instance be subgrouped based on their amount of cholesterol (Wang et al., 2006). Such factors may add to the intrinsic fusogenicity of the vesicles. Nevertheless, this is a poorly studied question.

The effective discrete release probability upon action potential stimulation is the probability for each vesicle to release given that the synapse is not silent. This can be calculated by dividing the charge for the evoked response by the charge for the total readily releasable pool (Rosenmund and Stevens, 1996). Another way of measuring the release probability is by tagging vesicles with fluorescent markers, measuring the peak fluorescence upon an evoked response and dividing it by the response obtained upon depletion of the readily releasable pool (Granseth and Lagnado, 2008). In glutamatergic hippocampal neurons the effective discrete release probability is commonly 3–10% and the average evoked release rate at 4 mM external calcium is 24 pools/s (Basu et al., 2007). A disadvantage of measuring the effective release probability is the loss of information about heterogeneity of vesicular release probability. For instance it has been suggested that action potential trains only release a subset of vesicles compared to sucrose stimulation in glutamatergic hippocampal neurons, but in GABAergic neurons both methods give similar amounts of release (Moulder and Mennerick, 2005). This indicates a higher heterogeneity in excitatory hippocampal neurons compared to inhibitory ones.

In whole-cell recordings the effective release probability will reflect the release from all vesicles that have fused, but it is important to remember that not all synapses release upon stimulation. The global fitness of a single synapse is reflected in the intrinsic release probability (*P*_*r*_), which lies between 20% and 70% in hippocampal neurons (Nadkarni et al., 2012). It has also been seen that multiple vesicles can release from the same release site upon a single action potential stimulation with a refractory time between 5 and 20 ms in hippocampal neurons (Stevens and Wang, 1995; Christie and Jahr, 2006; Nadkarni et al., 2010). It is possible that the wide range of this refractory time could account for different biophysical phenomena for instance site priming and the reluctance of the cell to undergo further fusion events at a critical point of plasma membrane tension. For spontaneous fusion, the frequency of events can be estimated directly from the post-synaptic response over time under resting conditions and has been seen to lie between 0.0005 and 0.001 vesicles per second in hippocampal synapses (Murthy and Stevens, 1999; Basu et al., 2007).

## Endocytosis and vesicle replenishment

So far we have seen that short-term plasticity is determined by recruitment steps of vesicles to the membrane, release probability as well as calcium dynamics and synapse architecture. After fusion it is also important that the cell and the individual active zones are in states where they can allow new vesicles to fuse. A factor that has been suggested to be important for the cell to allow fusion is membrane tension, since a critical number of endocytosis events are required for exocytosis. This can be an explanation for the long interstimulus interval required for consecutive fusion events at a single active zone discussed earlier (Watanabe et al., 2013). As mentioned above, for the active zones to allow new fusion events, it is important that t-SNAREs and any other cofactors are present, which are possibly replenished from the membranes of fused vesicles.

In addition to the biophysical state of the cell and the individual active zones, enough vesicles need to be available for the synapse to continue releasing neurotransmitters. Under depletion of vesicles by sustained high frequency stimulation, it becomes relevant from which source vesicles travel to the active zone and whether there are any vesicles available in this reserve source to replenish the readily releasable pool. This is, however, not a rate-limiting factor during short trains of high frequency stimulation. In hippocampal neurons two major vesicle replenishment pathways are known, namely (1) membrane retrieval directly from clathrin-mediated endocytosed vesicles and (2) vesicle recruitment from the reserve pool. However, other fast vesicle retrieval modes have also been suggested in hippocampal neurons as discussed below. It is important to note that vesicle retrieval does not only include the retrieval of the membrane, but also that proper vesicles need to be formed, acidified and refilled with neurotransmitters.

The different time scales of membrane retrieval modes can be studied by (1) FM dyes which can be used to measure at second scales, (2) synaptophluorins and quantum dots to measure at below second scale, and (3) membrane capacitance with a time resolution on the millisecond scale (Wu, 2004). By using these methods, both 10–100 s and 0.1–2 s time scales have been seen in hippocampal neurons (Ryan et al., 1993; Hallermann et al., 2003). Clathrin-mediated endocytosis takes place outside the active zone and operates at a time scale of about 15–30 s (Granseth and Lagnado, 2008; Hori and Takahashi, 2012; Watanabe et al., 2013). It was therefore suggested to be too slow to replenish vesicles and together with evidence from electron microscopy analysis gave rise to the kiss-and-run hypothesis (Ceccarelli et al., 1973; Gandhi and Stevens, 2003). This states that a vesicle touches the plasma membrane and releases its neurotransmitters within 1 s, but does not fuse fully with the plasma membrane. Thus, the vesicle could potentially be reused immediately, but it possibly needs to be reprimed molecularly, and depending on the time scales of the priming process kiss-and-run may or may not be an advantage at fast stimulation. Using quantum dot imaging, it has been suggested that kiss-and-run is used at the beginning of a high frequency stimulation train, and is overtaken later on by slow endocytosis (Zhang et al., 2009). Nevertheless, this was later contradicted using phlourines (Granseth et al., 2006). Recently, an alternative form of fast endocytosis of fully fused vesicles taking place within the time scales of 100 ms directly at the active zone has been suggested (Watanabe et al., 2013).

For vesicle recruitment from the reserve pool, STED microscopy data from hippocampal mass cultures suggest that the recycling pool constitutes 10–20% of all vesicle pools and that the reserve pool is immobile under low and medium stimulation conditions. In hippocampal slices, however, essentially all vesicles seem to be mobilized after 2–4 weeks in culture (Rose et al., 2013). However, after prolonged high-frequency stimulation the reserve vesicles seem to transform into recycling vesicles (Kamin et al., 2010). There is also evidence that the mobility of vesicles is higher after endocytosis than under rest (Kamin et al., 2010). The question of which mechanism the hippocampal readily releasable pool uses to become replenished under longer trains of high-frequency stimulation was addressed in a model of hippocampal synapses, where the authors included replenishment both directly by clathrin-mediated endocytosis and from the reserve pool (Granseth and Lagnado, 2008). The model predicted that most RRP vesicles were replenished by clathrin-mediated endocytosis. It has also been suggested that clathrin-mediated endocytosis is modulated by calcium influx from voltage-gated calcium channels and calmodulin activation, which is an important factor for recovery from STD (Wu et al., 2014).

## Neurotransmitter filling and quantal size

In order to have a reserve recycling pool, the retrieved vesicles need to be filled with neurotransmitters. To retrieve released neurotransmitters from the synaptic cleft, the plasma membrane uses *N*a^+^ and *C*l^−^ dependent plasma membrane transporters. Excitatory amino acid transporters, which are used to take up glutamate are usually found on astrocytes, but also neuron specific subtypes exist (Blakely and Edwards, 2012). After the glutamate has been converted to glutamine, the astrocytes transfer them to neurons. Upon exocytosis, the synaptic vesicle loses its acidic pH and needs to be reacidified in order to build up a gradient for neurotransmitter filling. Vesicles are filled by neurotransmitters with the help of proton pump ATPase coupled neurotransmitter channels. Hence, how much neurotransmitter a vesicle can fill and the rate of filling are determined by its pH or its membrane potential. In glutamatergic neurons where the membrane potential is the more important determinant, the gradient for refilling is counteracted by chloride (Omote et al., 2011). In GABAergic neurons, however, the pH gradient is the dominating contributor (Blakely and Edwards, 2012).

The amount of neurotransmitter per vesicle is referred to as the quantal size, and it is known to vary between synapses (Bekkers et al., 1990). The quantal size can be measured in terms of charge by integrating the current from spontaneous release, and by looking at the distribution of current amplitudes. One interesting question is whether fast endocytosis could potentially influence the quantal size since the fusion pore is small and transient. In cultured hippocampal synapses, average quantal size has been seen not to be affected by transient fusion (Wu, 2004). Another interesting question is how wide the spread of quantal sizes looks in a population of individual synaptic vesicles. The distribution of quantal size is often Gaussian in the neuromuscular junction, although it is often skewed in central synapses (Auger and Marty, 2000).

The quantal size is directly regulated by the reacidification and neurotransmitter filling steps of the vesicle as seen above. The time constant for refilling was earlier thought to be on a minute scale, but was recently measured to be 15 s by depleting the glutamate from recycling vesicles and measuring the EPSC response rate upon consecutive stimulations upon glutamate uncaging in the Calyx of Held (Hori and Takahashi, 2012). The time constant for reacidification has previously been dissected from the refilling rate by trapping pHluorin tagged vesicles and monitoring at what speed they are reacidified. Speed of reacidification occurs with a time constant of 5.65 s at 25°C (Granseth and Lagnado, 2008).

## Concluding remarks

In this review we have discussed what information electrophysiology, imaging and mechanistic models have given on kinetic and quantitative biophysical properties that underly short-term plasticity in hippocampal neurons. With this we want to highlight that the underlying mechanisms of neurotransmitter release are multifaceted, and that although mechanistic models cannot always catch the full complexity of the nature of neurotransmitter release, they can be useful as integrators of different types of data and to account for the interactions of the biophysical mechanisms. Considering the broad range of time scales involved in the underlying biophysical mechanisms, it is clear that different mechanisms become more important in different cases. For short-term plasticity at time scales below 10 s, the fusogonicity of vesicles and their recruitment rates are especially important factors. However, the relative impact of these mechanisms requires further investigation.

### Conflict of interest statement

The authors declare that the research was conducted in the absence of any commercial or financial relationships that could be construed as a potential conflict of interest.
